# Novel Sequence Types of *Listeria monocytogenes* of Different Origin Obtained in the Republic of Serbia

**DOI:** 10.3390/microorganisms9061289

**Published:** 2021-06-12

**Authors:** Tatiana Yu. Bespalova, Tatiana V. Mikhaleva, Nadezhda Yu Meshcheryakova, Olga V. Kustikova, Kazimir Matovic, Marko Dmitrić, Sergey S. Zaitsev, Maria A. Khizhnyakova, Valentina A. Feodorova

**Affiliations:** 1 Federal Research Center for Virology and Microbiology, Branch in Samara, 443013 Samara, Russia; 27bt@mail.ru (T.Y.B.); tatyanamihaleva@mail.ru (T.V.M.); nadya.meshcheryakova.90@mail.ru (N.Y.M.); o_v_kus@mail.ru (O.V.K.); 2 Department for Laboratory Diagnostic, Veterinary Specialized Institute Kraljevo, 36000 Kraljevo, Serbia; matovic@vsikv.com (K.M.); dmitric@vsikv.com (M.D.); 3 Department of Food Safety, Veterinary Specialized Institute Kraljevo, 36000 Kraljevo, Serbia; 4 Federal Research Center for Virology and Microbiology, Branch in Saratov, 410028 Saratov, Russia; zaytsev-sergey@inbox.ru (S.S.Z.); khizhnyakova_mariya@mail.ru (M.A.K.)

**Keywords:** *Listeria monocytogenes*, isolates, food, ruminant, multi-locus sequence typing, MLST, sequence type, ST, housekeeping genes, clonal complex, phylogenetic lineage, internalins

## Abstract

*Listeria monocytogenes*, the causative agent of listeriosis, is amongst the major food-borne pathogens in the world that affect mammal species, including humans. This microorganism has been associated with both sporadic episodes and large outbreaks of human listeriosis worldwide, with high mortality rates. In this study, the main sequence types (STs) and clonal complexes (CCs) were investigated in all of the 13 *L. monocytogenes* strains originating from different sources in the Republic of Serbia in 2004–2019 and that were available in the BIGSdb-Lm database. We found at least 13 STs belonging to the phylogenetic lineages I and II. These strains were represented by ST1/ST3/ST9 of CC1/CC3/CC9, which were common in the majority of the European countries and worldwide, as well as by eight novel STs (ST1232/ST1233/ST1234/ST1235/ST1238/ST1236/ST1237/ST1242) of CC19/CC155/CC5/CC21/CC3/CC315/CC37, and the rare ST32 (clonal complex ST32) and ST734 (CC1), reported in the Republic of Serbia, the EU, for the first time. Our study confirmed the association of CC1 with cases of neuroinfection and abortions among small ruminants, and of CC3 and CC9 with food products of animal origin. The strains isolated in 2019 carried alleles of the internalin genes (*inlA/inlB/inlC/inlE*) characteristic of the most virulent strains from the hypervirulent CC1. These findings demonstrated the genetic relatedness between *L. monocytogenes* strains isolated in the Republic of Serbia and worldwide. Our study adds further information about the diversity of the *L. monocytogenes* genotypes of small ruminants and food products, as the strain distribution in these sources in Serbia had not previously been evaluated.

## 1. Introduction

*Listeria monocytogenes* is a gram-positive bacterium and the causative agent of listeriosis, a zoonotic disease that affects mammal species, including humans [1,2]. *L. monocytogenes* is amongst the major food-borne pathogens in the world and has attracted much research attention from public health, veterinary medicine, and food authorities over the recent decades [3]. *L. monocytogenes* has been linked to sporadic episodes, as well as large outbreaks, of human listeriosis worldwide [4,5]. Although relatively rare (the annual incidence rate ranges from 0.2 to 0.5 cases per 100,000 population), listeriosis is responsible for high hospitalization rates of over 95% and mortality rates (average fatality rates are estimated to be 20–30% of hospitalized patients and can reach 40% in outbreaks related to foodborne infections) [5,6]. For example, within the European Union, the case fatality for *L. monocytogenes*-associated infection stood at 15.6% in 2018 [7].

The main clinical manifestations of listeriosis in both humans and animals (it is of particular relevance to ruminants) include gastroenteritis, septicemia, and more frequently, abortion, and central nervous system (CNS) infections such as meningitis, meningoen-cephalitis, and rhombencephalitis [6,8,9]. CNS involvement is a characteristic feature and accounts for the high mortality associated with listeriosis [6]. The identical neuropathology of listeric rhombencephalitis in humans and ruminants indicates that neurotropic strains common to both hosts are responsible for the development of the disease [6]. The results of Dell’Armelina Rocha et al. [8] support the hypothesis that ruminants represent possible natural reservoirs of *L. monocytogenes* strains capable of causing epidemics of listeriosis in humans.

In both humans and ruminants, infection with *L. monocytogenes* occurs predominantly due to the consumption of contaminated feed and food (including dairy products, meat, fish, and vegetables), as well as through different food production processes [9]. The persistence of *L. monocytogenes* in food processing environments is still considered to be the major source of ready-to-eat (RTE) food contamination [10].

Today, an increasing rate of listeriosis has been reported in several European countries and other continents. Thus, outbreaks caused by *L. monocytogenes* occurred; (i) in Switzerland, associated with Tome cheese and with the use of semi-hard cheeses produced by Käserei Vogel AG; (ii) in the United Kingdom associated with sandwich consumption; (iii) in the Czech Republic associated with soft cheese; and (iv) a recent multinational outbreak was due to the consumption of Quargel cheese in Austria and Germany [4,11]. Recently, in 2020, several outbreaks of listeriosis were also registered in the United States. They were associated with enoki mushrooms (winter pollen, Flammulina velutipes) produced in the Republic of Korea (36 cases in 17 states, 4 deaths). More recently, a progressive increase in listeriosis cases was noted from mid-June 2017 in South Africa, heralding what was to become the world’s largest listeriosis outbreak during the last decade. In fact, a total of 1060 cases were reported for the period from 1 January 2017 to 17 July 2018. *L. monocytogenes* microorganisms were found in environmental sampling swabs of the production facility and in ready-to-eat processed meat products [12]. As such, listeriosis is considered to be one of the greatest global burdens among foodborne diseases worldwide [13].

Multilocus sequence typing (MLST) is a reference method for the global epidemiology and population biology of bacteria, and its application to *L. monocytogenes* allows effectively isolating comparisons across laboratories in open accessible databases, such as the one provided by Institute Pasteur (https://bigsdb.web.pasteur.fr/listeria/listeria.html, accessed on 11 June 2021) [14,15,16].

In the last decade, the characterization of genetic profiles through the use of molecular methods has helped to track and demonstrate the genetic diversity among *L. monocytogenes* isolates obtained from various sources. Strains can be compared on a global level and this helps to track forward and trace backward pathogen contamination events in food processing facilities and in outbreak scenarios [15].

In *L. monocytogenes* MLST, the sequences of seven housekeeping genes (length 399–537 bp), which are spread across dispersed genomic locations, are amplified by PCR using universal primers before sequencing. The unique alleles are allocated to allelic profiles, and subsequently the sequence type (ST) is determined [15,16]. Generally, the major advantage of MLST is the ability to reconstruct ancestral and evolutionary linkages between *L. monocytogenes* isolates. MLST detects all genetic variations within the amplified housekeeping genes that accumulate slowly.

The initial *L. monocytogenes* MLST scheme was published by Salcedo et al. [17], the actual MLST protocol refers to Ragon et al. [18], who published the phylogenetic structure of 360 *L. monocytogenes* representatives by MLST typing.

By MLST, *L. monocytogenes* are divided into four phylogenetic lineages: the majority of *L. monocytogenes* isolates cluster into two lineages (I and II). Two additional lineages (III and IV) have subsequently been identified, are rarely isolated, and mostly occur in ruminants [19,20].

The majority of STs can be assigned to one of the seven major clones or clonal complexes (CC). The predominant CCs were CC1, CC2, and CC3 (lineage I). Lineage II was more heterogeneous and comprised different CCs [16,21].

Currently, the distribution of *L. monocytogenes* STs in different world regions is being intensively studied. However, for some countries only limited information on the diversity of *L. monocytogenes* genotypes is available. In addition, there is relatively little information on the sequences of surface and secreted proteins by *L. monocytogenes* from the internalin family, which are the main essential virulence factors allowing *L. monocytogenes* to cross the intestinal barrier in order to invade the epithelial and endothelial cells of the host [1].

Recently, the initial *L. monocytogenes* MLST scheme was successfully supplemented with an analysis of the genes expressing the proteins of a protein family known as the internalins, which contribute to the virulence of this microorganism-multi-virulent-locus sequence typing (MvLST) [22]. MLST and MvLST are considered great tools for studying *L. monocytogenes* genotype distribution among animal and human clinical cases, foods, and other sources, as well as for unraveling food-borne outbreaks [2,15].

**Figure 1:**
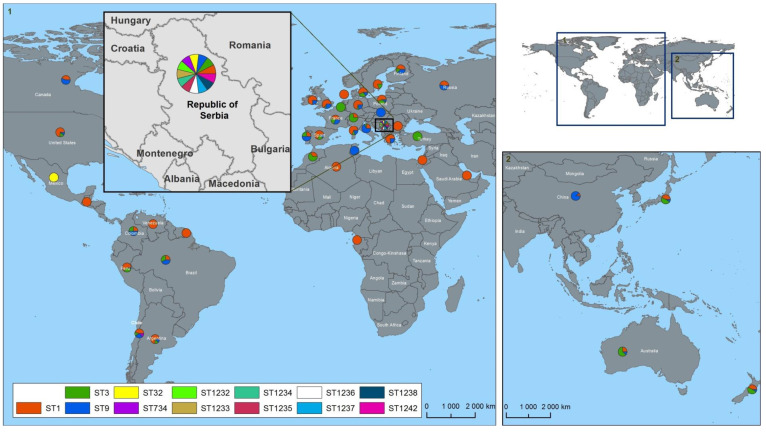
Location of the study area. The territory of the Republic of Serbia is illustrated in a separate square. The *L. monocytogenes* strains of different STs are highlighted in the colored circles: red—ST1, green—ST3, blue—ST9, yellow—ST32, plum—ST734, lime—ST1232, brown—ST1233, teal—ST1234, maroon—ST1235, white—ST1236, azure—ST1237, hunter green—ST1238, and hot pink—ST1242.

The Republic of Serbia is a country in central and southeastern Europe, in the southern Pannonian Plain and central Balkans. It borders Hungary in the north, Romania in the northeast, Bulgaria in the southeast, North Macedonia in the south, Croatia and Bosnia and Herzegovina in the west, and Montenegro in the southwest (Figure 1). According to the report on infectious diseases by the Institute of Public Health of Serbia (Belgrade), a small number of cases of human listeriosis are registered in the Republic of Serbia, reflecting the sporadic nature of invasive listeriosis in this country. From 2014 to 2018, the annual incidence rate ranged from 0.04 to 0.19 cases per 100,000 population, but the mortality rate in 2018 was 12.5% [23]. In the Republic of Serbia to this day, *L. monocytogenes* have only been studied in food, the food industry environment, and humans, and, unlike some neighboring countries, there is limited knowledge about their genetic characteristics, distribution, and effects, especially on ruminant populations, and information about the veterinary significance of *L. monocytogenes* is still insufficient. In fact, no data have been reported on the distribution of *L. monocytogenes* STs there, although the Republic of Serbia is one of the European countries with the largest population of small ruminants.

The aims of this study were: (a) to reveal genetic relationships of STs among the *L. monocytogenes* strains in the Republic of Serbia and worldwide by MLST technique; and (b) to determine the partial sequences of *L. monocytogenes* virulence genes which encode the proteins of the internalin family (namely, *inlA*, *inlB*, *inlC* and *inlE*), making them adhere and invade host cells [22,24,25,26], by MvLST analysis.

## 2. Materials and Methods

### 2.1. L. Monocytogenes Strains and Culture Cultivation

A total of five *L. monocytogenes* strains were obtained from the Veterinary Specialized Institute Kraljevo (Kraljevo, the Republic of Serbia) for further analysis. The isolates were collected throughout 2019 from animals which had died of *listerial neuroinfection* (postmortem sectional material), fetus of small ruminants from farms from the Republic of Serbia, and from food and food production environment (Table S1, Figure 1, Supplementary Materials), and designated as Serbia-2019 isolates. The strains were kept frozen at −70 °C. The bacterial cultures were plated on brain–heart infusion (BHI) (BD Bioscience, EU) agar and grown overnight before an experiment.

Additionally, eight *L. monocytogenes* strains (118/2004, 124/2004, 2, 265/2010, 2184/2010, 2960/2012, 4250/2009, 22/2008) that were detected in the Republic of Serbia in human clinical samples and food specimens earlier, in 2004–2012 (Serbia-2004–2012) (https://bigsdb.pasteur.fr/listeria/listeria.html, accessed on 14 December 2016 for 118/2004, 124/2004, 2, 265/2010, 2184/2010, 2960/2012 and 4250/2009; January, 02, 2017 for 22/2008), were also included in this study (Table S1, Supplemental Data 1).

### 2.2. DNA Extraction

Lysates of overnight *L. monocytogenes* cultures of Serbia-2019 were obtained as previously described, with modifications [27]. Then, 100 µL of a 24-h culture grown in heart–brain broth (Difco, Detroit, MI, USA) at a temperature of 37 °C was resuspended in a pre-lysis buffer of the following composition: 20 mM Tris (hydroxymethyl) aminomethane (Tris)/hydrochloric acid (HCl); 2 mM EDTA ethylenediaminetetraacetic acid (EDTA); 1% Triton X-100; pH 8; all components (Sigma-Aldrich, St. Louis, MO, USA) containing 20 mg/mL were added directly before the use of lysozyme (50 mg/mL) (Serva, Heidelberg, Germany). After the incubation of the mixture for 1 h at a temperature of 37 °C, 1 µL proteinase K (10 mg/mL, Sigma) was added and incubated for 1 h at a temperature of 56 °C. The lysates were boiled for 10 min to inactivate proteinase K and stored at a temperature of 4 °C.

### 2.3. PCR

The PCR was run with 1 µL of lysate in the CFX96 amplifier (Bio Rad, Hercules, CA, USA). An Encyclo Plus PCR kit (Evrogen, Moscow, Russia) and primers (Syntol, Moscow, Russia) were used for DNA amplification (Supplementary Materials).

### 2.4. Multi-Locus Sequence Typing (MLST) and Multi-Virulent-Locus Sequence Typing (MvLST) Analysis

The MLST scheme based on the sequences of seven housekeeping genes (*abcZ*, bglA, *cat*, *dap*, *dat*, *ldh,* and *lhkA*) was used. For this purpose, a panel of primers was successfully applied [17,18] (Supplemental Data 1). The relevant target housekeeping gene fragments were amplified as previously described [14,28], with minor modifications. The following temperature conditions were used: 94 °C, 2 min; (92 °C, 30 s; 55 °C, 30 s; 72 °C, 2 min) × 30; 72 °C, 10 min; the number of cycles was lowered from 35 to 30; melting temperatures (Tm) were lowered to 48 °C for *dat*.

For the MvLST, the PCR amplification of the internal fragments of four internalin genes (*inlA, inlB, inlC*, and *inlE*) (Supplemental Data 1) for virulence gene analysis was performed as previously described [1,28], with modifications for *inlB,* in which the following parameter was changed: Tm = 48 °C.

PCR products were evaluated by electrophoresis in 1% agarose gel. Amplified DNA was eluted from the reaction mixture using Cleanup Standard (Evrogen, Moscow, Russia) according to the manufacturer‘s instructions. Verification on 1.5% agarose gel followed, after which it was sent for sequencing to the “Genome” center (http://www.genome-center.ru, accessed on 4 August 2020).

### 2.5. PCR Product Sequencing

Individual PCR product sequencing was performed using a set of reagents ABI PRISM ^®^ BigDye ^™^ Terminator v. 3.1, followed by the analysis of the reaction products on an automatic sequencer, Applied Biosystems 3730 DNA Analyzer (Applied Biosystems, Foster City, CA, USA). Electrophoretic DNA separation was performed in 50-cm capillaries with POP7 polymer.

All the “house-keeping gene”-derived sequences were deposited in the Institute Pasteur MLST BIGSdb-Lm database (https://bigsdb.pasteur.fr/listeria/primers_used.html, the access numbers: 76379-76383, accessed on 22 March 2021).

The identification of obtained sequences was executed with the help of the BIGSdb-Lm database (https://bigsdb.pasteur.fr/listeria/primers_used.html, accessed on 22 March 2021) and included a search for allele numbers, relevant sequence type (ST), clonal complex (CC), and phylogenetic lineage for each *L. monocytogenes* isolate.

The determination of allelic profiles for each consensus gene sequence of internalins derived from Serbia-2019 strains was carried out by means of comparing the relevant sequences against loci lmo0433 (*inlA*), lmo0434 (*inlB*), lmo1786 (*inlC*), and lmo0264 (*inlE*) of the reference nucleotide sequences of *L. monocytogenes* strains that were available in the BIGSdb-Pasteur MLST database (https://bigsdb.pasteur.fr/listeria/listeria.html, accessed on 4 April 2021).

### 2.6. Phylogenetic and Data Analysis

The sequences were proofread and assembled in Chromas Lite MFC Application version 2.1.1.0. (https://technelysium.com.au/wp/, accessed on 22 March 2021). The alignment of multiple concatenated sequences was carried out with the multiple alignment tool MultAlin (http://multalin.toulouse.inra.fr/multalin/, accessed on 22 March 2021).

A minimum-spanning tree from allelic profiles based on MLST loci scheme was generated and visualized using GrapeTree [29], in order to demonstrate the phylogenetic relationships between the Serbia-2019 isolates and *L. monocytogenes* strains (118/2004, 124/2004, 2, 265/2010, 2184/2010, 2960/2012, 4250/2009, 22/2008) that were detected in either the Republic of Serbia in 2004–2012 (Serbia-2004–2012) or other world regions and showed identical STs and CC (Table S1, Supplemental Data 1).

The phylogenetic trees were constructed using the Maximum Likelihood algorithm with 1000 bootstrap replicates. The concatenated sequences of 27 reference representative *L. monocytogenes* isolates from the BIGSdb-Pasteur MLST database (https://bigsdb.pasteur.fr/listeria/listeria.html, accessed on 19 May 2021) (Supplemental Data 1) were aligned and analyzed in MEGA 7 [30].

Allelic numbers and STs were determined using the *L. monocytogenes* BIGSdb-Pasteur MLST database (https://bigsdb.pasteur.fr/listeria/listeria.html, accessed on 19 May 2021). Nucleotide diversity was analyzed with DnaSP version 6 [31].

The results of MLST typing are now available in the BIGSdb-*Lm* MLST database (http://www.pasteur.fr/mlst, accessed on 11 June 2021) with IDs, as indicated in Table 1.

### 2.7. Cartographic and Phylogeographic Analysis

The distribution of *L. monocytogenes* ST1, ST3, ST9, ST32, ST734, and ST1232-ST1238, ST1242, which are available in the BIGSdb-Pasteur MLST database (https://bigsdb.pasteur.fr/listeria/listeria.html, accessed on 11 June 2021) for spatial data processing and visualization in the Republic of Serbia and worldwide was performed using the ESRI geographic information system ArcGIS 10.6.1 (Esri, Redlands, CA, USA) and the DSW electronic map of the world (the Russian Federation). To display the multiple STs on the map, a color pie chart tool (http://www.esri.com/software/arcgis/arcview/index.html, accessed on 21 May 2021) was used.

## 3. Results and Discussion

In this study, the polymorphism in the housekeeping genes of all 13 *L. monocytogenes* strains that were isolated during the routine monitoring to control of the spread of listeriosis in the Republic of Serbia in 2004–2019 was carefully examined. Initially, five *L. monocytogenes* strains isolated recently, in 2019, from the Republic of Serbia were carefully investigated using MLST and MvLST techniques [14,17,18]. Among them three strains were derived from food-associated samples and two isolates had an animal origin (Table S1, Supplemental Data 1). By MLST, five different STs, namely ST1, ST3, ST9, ST32, and ST734, were revealed. Only two STs, ST1 and ST734, belonged to the identical CC1 while the other STs corresponded to three different clonal complex CCs: (i) ST3–CC3, (ii) ST9–CC9, and (iii) ST32–ST32, respectively. Overall, all the STs were assigned to two phylogenetic lineages, I and II. In fact, the genetic lineage I was prevalent, as the majority of STs (4/5, 80%) were represented by the relevant ST1, ST3, ST32, and ST734. Only a single ST9 (1/5, 20%) could be allocated to the genetic lineage II.

**Figure 2:**
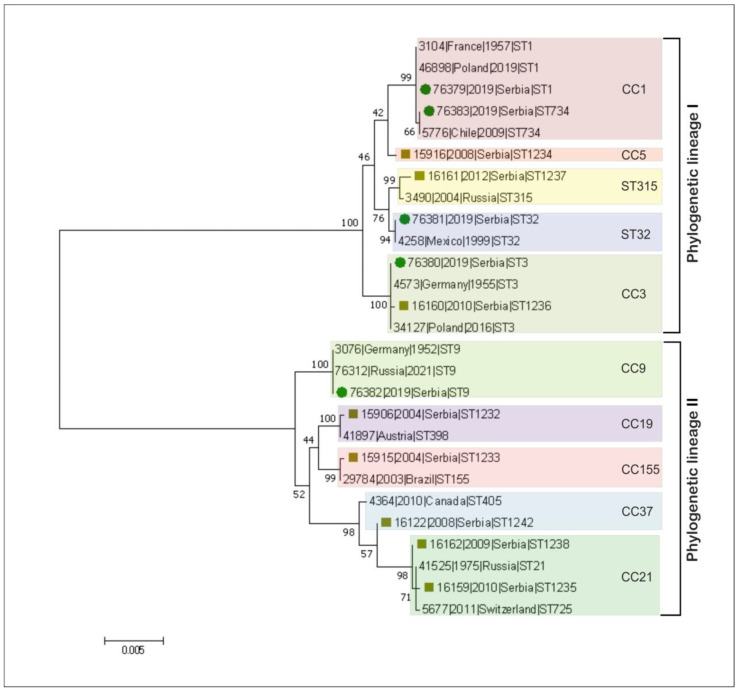
Molecular phylogenetic analysis based on the representative *L.* monocytogenes concatenated seven housekeeping gene fragments showed the position of strains isolated in the Republic of Serbia in 2019 (green dot) in relation to strains previously isolated in either this country in 2004–2012 (yellow square) or other parts of the world and in which the identical STs and CCs were found. The concatenated sequences were aligned and analyzed in MEGA7 [30]. The trees were constructed using the maximum likelihood algorithm with 1000 bootstrap replicates.

Molecular phylogenetic analysis based on the MLST data obtained, showed that each of the Serbia-2019 strains was clearly clustered with the reference *L.* monocytogenes organisms, possessing the same STs and isolated earlier in either the Republic of Serbia or worldwide (Figure 2). In addition, we demonstrated that two STs, ST734 and ST32, and the relevant clonal complexes, CC1 and ST32, detected among Serbia-2019 strains were found in Europe for the first time (Figure 2).

Importantly, only a single strain (#76380/DP 1093/2019) from Serbia-2019 belonged to CC3 that had also previously been revealed in the Republic of Serbia in humans with L. monocytogenes-associated infection (in 2010, #16,160/2184/2010), while the rest, CC1, CC9, and ST32, were recognized here for the first time. However, both CC3 strains had different STs, namely, ST3 for the organism detected in 2019, and ST1236 for the strain isolated in this country in 2010. These strains demonstrated identical alleles, with six out of the seven loci found in ST3 and ST1236, respectively, and they differed by a single allele ldh (Table S1, Supplemental Data 1). Although these strains originated from different sources, food and human specimens, they formed a single cluster, indicating that both isolates could have a common ancestor (Figure 2).

In contrast to Serbia-2019, six of seven CCs revealed in the Republic of Serbia during 2004–2012 were represented by other clonal complexes differing from those found by us in this country in 2019. Additionally to CC3 (ST1236), there were six different clonal complexes, namely CC5, CC19, CC21, CC37, CC155, and CC315 of seven different STs, such as: ST1234, ST1232, ST1235 and ST1238, ST1242, ST1233 and ST1237, respectively. All these STs were also assigned to two phylogenetic lineages, I and II. However, in contrast to Serbia-2019 strains, the phylogenetic lineage II was prevalent (5/8, 62.5%, CC19-ST1232, CC21-ST1235, CC21-ST1238, CC37-ST1242, and CC155-ST1233, respectively) compared with the phylogenetic lineage I (3/8, 37.5%, CC3-ST1236, CC5-ST1234, and CC315-ST1237) (Table S1, Supplemental Data 1). In fact, the majority of the Serbia-2004–2012 strains were derived from human clinical cases of listeriosis (ST1232, ST1233, STs1235–1238, and ST1242) except the only ST1234 (CC5), which was detected in a food specimen (Table S1, Supplemental Data 1).

**Figure 3:**
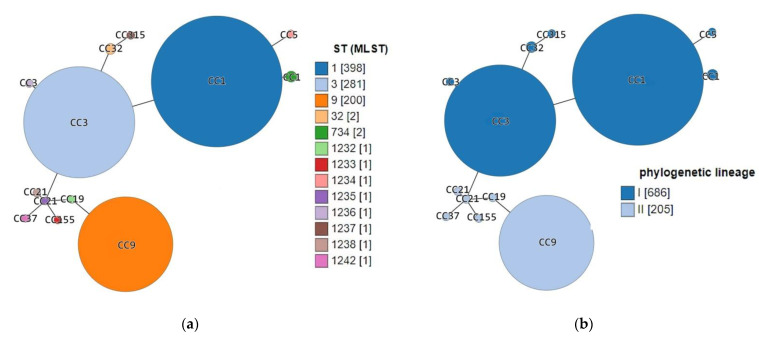
Minimum spanning tree demonstrating the genetic relatedness between *L. monocytogenes* strains isolated in the Republic of Serbia in 2004–2012 and 2019 and worldwide (only ST1, ST3, ST9, ST32, and ST734 are included). Each individual circle represents either (**a**) single clonal complex CC or (**b**) one sequence type (ST). The circle’s sizes are proportional to the number of strains. Links between circles represent the number of allelic mismatches between either (**a**) CCs or (**b**) STs (Supplemental Data 1).

**Figure 4:**
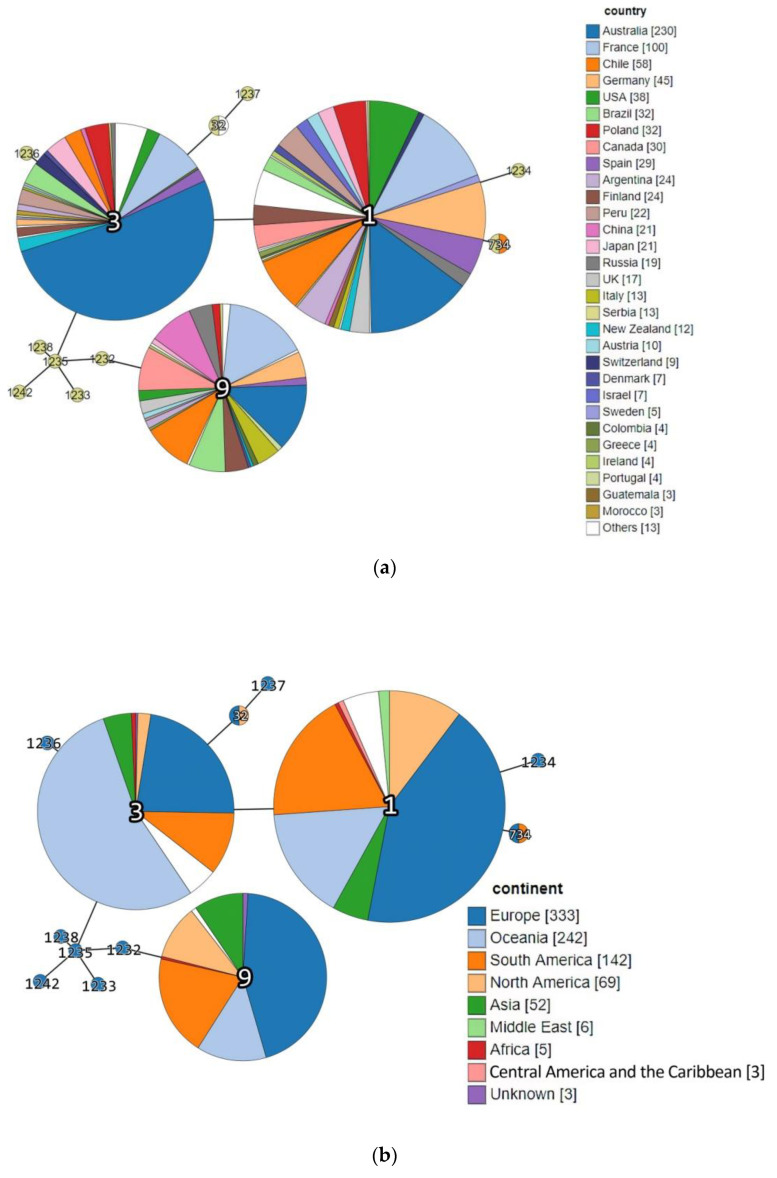

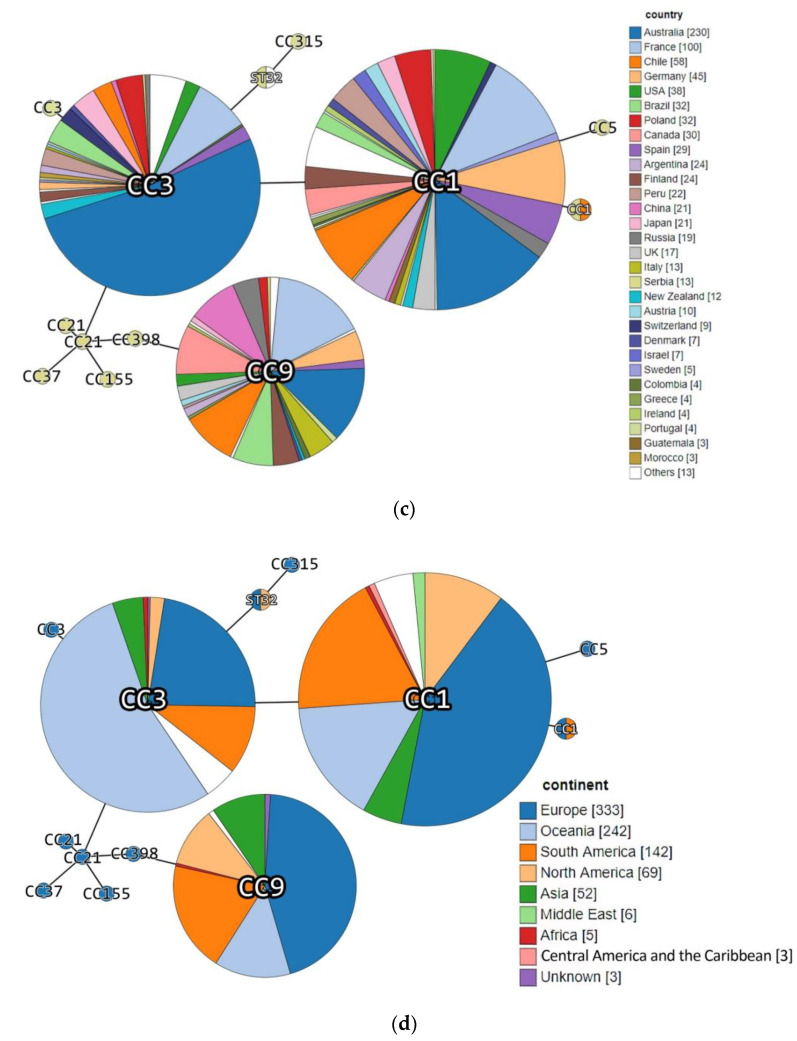
Minimum spanning tree demonstrating genetic relatedness between *L. monocytogenes* strains isolated in the Republic of Serbia (in 2004–2012 and 2019, respectively) and other countries. Each individual circle represents either: (**a**,**c**) single country or (**b**,**d**) a certain part of the world. The circle sizes are proportional to the number of strains. Links between circles represent the number of allelic mismatches between either (**a**) individual countries or (**b**) different parts of the world (Supplemental Data 1).

Interestingly, previous epidemiologic studies demonstrated that lineage I is generally overrepresented among human clinical listeriosis cases, while lineage II strains were found to be major contaminants of food products [5,9,19]. However, in fact, among the Serbia-2019 strains, non-human clinical isolates of animal and food origin belonging to lineage I were first detected (Table 1). Nevertheless, the phylogenetic tree constructed based on MLST data proved the phylogenetic relatedness between *L*. *monocytogenes* strains detected in the Republic of Serbia in 2019 and more recently, in 2004–2012 (Figure 2). Moreover, a minimum spanning tree showed a real genetic relatedness between Serbia-2019 and all the *L. monocytogenes* strains (Serbia-2004–2012), belonging to either the relevant STs and CCs (ST1, ST3, ST9, ST32, ST734 and CC3, CC5, CC19, CC21, CC37, CC155 and CC315) (Figure 3a) or STs and phylogenetic lineages (I and II) (Figure 3b) that were isolated earlier worldwide (Figure 4a,b). These findings could be correlated with the observations of previous studies suggesting that ruminants and food could represent a possible source of human listeriosis [8,9,10].

The majority of the L. monocytogenes strains belonging to ST1, ST3, and ST9 that were similar to the Serbia-2019 isolates of the identical STs (ST1, # 76379/DP 9228/2019, ST3, #76380/DP 1093/2019 and ST9, #76382/DP 7675/2019, respectively) were regularly detected in Australia (*n* = 230), France (*n* = 100), Chile (*n* = 58), Germany (*n* = 45), the USA (*n* = 38) etc. (Figure 4a). These STs were more often found in Europe, Oceania, and South America, rarely in North America and Asia, and occasionally in the Middle East, Africa, Central America, etc. (Figure 4b). Both ST1 and ST9 had been occasionally isolated in some neighboring countries such as: Bulgaria (1965) and Greece (1959) from animal specimens, and Greece (2018) from human samples (Supplemental Data 1) as well. No ST3 (CC3) was detected in the countries neighboring the Republic of Serbia, whereas since 1955–1957 the ST3 strains have been widely isolated in other European countries, namely France, Spain, Poland, Germany, Finland, Russia, Italy, Austria, Switzerland, Denmark, Sweden, Greece, Ireland, Portugal (Figure 4a, Supplemental Data 1). Interestingly, CC3 is the fourth most common clonal complex in Europe [16], and is predominant in Australia and has been detected in South and North America, Japan, and Oceania. The data obtained correlated with the observations of Parisi et al. [21] on the worldwide distribution of large clonal complexes CC1, CC3, and CC9 and the phylogenetic lineages I and II.

Surprisingly, two STs, ST734 and ST32, belonging to the clonal complexes, CC1 and ST32, and detected among Serbia-2019 strains were found in Europe for the first time. Thus, each of these STs and CCs were revealed only once, ST32 in Mexico, North America in 1999 (#4258/LM88502, clonal complex ST32) and ST734 in Chile, South America in 2009 (#5776/L-309-09, clonal complex CC1) *(*Figure 4a–d, Supplemental Data 1). Seemingly, L. monocytogenes strains circulating in the Republic of Serbia may possess more genetic diversity than those in some other European countries.

Indeed, at least eight novel STs (*ST1232-ST1238 and ST1242*) were only detected in the Republic of Serbia, in 2004–2012, and have not yet been found worldwide (Table S1, Figure 4, Supplemental Data 1). This indicates the possible presence of some undetectable sources and hidden reservoirs of *L. monocytogenes* in this territory.

**Figure 5:**
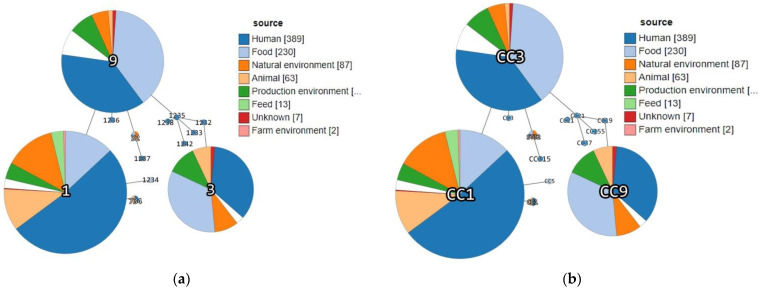
Minimum spanning tree demonstrating genetic relatedness between *L. monocytogenes* strains isolated in the Republic of Serbia in 2004–2012 and 2019, respectively, from animal, human, food, and environmental sources. Each individual circle represents either (**a**) one sequence type (ST) or (**b**) single clonal complex CC. The circle’s sizes are proportional to the number of strains. Links between circles represent the number of allelic mismatches between either (**a**) CCs or (**b**) STs (Supplemental Data 1).

Source category analysis showed a certain association between the sample origin and the ST and CC identified. Thus, Serbia-2019 strains belonging to CC1 (ST1 and ST734) were isolated from animal specimens, while CC3 and CC9 were derived from food sources, similarly to CC1 (ST1), CC3 (ST3), and CC9 (ST9) that had been detected earlier worldwide and principally had the same origin, from animal and food-associated isolates (Figure 5). In fact, the clonal complexes CC1 and CC3 were represented by the largest number of strains, being recognized as the most common globally prevalent isolates [29].

The majority of STs and CCs of *L. monocytogenes* strains isolated in the Republic of Serbia earlier, in 2004–2012, originated from human specimens (ST1236, ST1232, ST1235, ST1238, ST1242, ST1233 and ST1237, and CC3, CC19, CC21, CC37, CC155 and CC315, respectively) while only one of them (CC5, ST1234) was from a food source (Table S1, Supplemental Data 1).

Another significant finding of our research was the detection of ST1 belonging to CC1 and the phylogenetic line I (DP 9228/2019, Table S1, Supplemental Data 1) in the brain of dead sheep with listerial neuroinfection. This is consistent with the data of European researchers, who reported neurotropism and a strong association of ST1 with rhombencephalitis in ruminants which died during listeriosis outbreaks [2,32,33].

It is known that L. monocytogenes CC3 and CC9 are more often isolated in highly immuno-compromised patients [2,26] (Figure 5) and who were predominantly associated with food production sectors [26,34]. In our research, ST3 and CC3 and ST9 and CC9 was successfully isolated from cheese with prosciutto and smoked pork ribs (*DP 1093/2019,* DP 7675/2019, Table S1, Supplemental Data 1), respectively, which are related to meat-containing products. Our result correlates with many studies that have shown a high adaptability of ST9 isolates to meat production conditions. An overview of the diversity of strains circulating at all levels of the pork production chain in France showed that CC9 was directly associated with finished meat products [35]. This type of sequence was often found to be dominant in raw pork meat and usually preserved in slaughterhouses, meat processing plants, and meat products [36]. Thus, L. monocytogenes persistence in food processing environments (FPEs) increases the risk of food contamination and represents a major concern for the food industry and food safety [3,36,37].

Interestingly, the rare ST32 clonal complex ST32 showed an identical source (Production environment) when it was found in both cases, namely in the Republic of Serbia in 2019 and in Mexico in 1999. In contrast, the second rare CC1 ST734 was isolated in the Republic of Serbia from animal specimens, whereas the first isolate from Chile, 2009, was derived from a human sample (Supplemental Data 1). Notably, ST734 from Serbia-2019 was isolated from an aborted goat fetus and belonged to the hypervirulent clonal complex CC1 that was earlier detected among the *L. monocytogenes*-associated cases of abortion in ruminants in European countries (Slovenia, France, Great Britain, and Switzerland) [32]. It is quite possible, that this could be explained by the presence of internalins in the *L. monocytogenes* strains, especially the *InlA* protein that is required to cross the human maternofetal barrier and to cause fetal infection [38]. Importantly, ST32 detected among Serbia-2019 strains was obtained from the specimen derived from a fruit sorting machine (Table S1, Supplemental Data 1). Fruit processing facilities are reported to be potential sources of persisting *L. monocytogenes* contamination [39]. The 2014–2015 U.S. nationwide outbreak of listeriosis linked to apples was the first implication of fruit processing in the outbreaks of *L. monocytogenes*-associated food-borne illnesses [40]. In fact, 35 outbreaks of this listerial infection were identified in 12 US states from October 2014 to February 2015; 34 (97%) listeriosis patients were hospitalized and seven (20%) died. According to MLST analysis, environmental samples from the grower’s packing facility and distribution-chain apples yielded outbreak-related *L. monocytogenes* isolates. These observations highlighted the importance of minimizing fruit processing facility contamination with *L. monocytogenes* [41]. In addition, the MvLST method showed the presence of alleles of the internalin gene (*inlA1*), which is one of the main characteristics of the most virulent strains from the CC1 and CC2 clones [1,32] in the relatively rare sequence type ST32, which did not belong to any of the clonal complexes associated with outbreaks of listeriosis in both farm animals and humans.

Finally, internal fragments of the internalin genes, such as *inlA*, *inlB*, *inlC*, and *inlE,* were carefully studied to supplement the MLST data for the Serbia-2019 strains with virulence gene analysis, according to recommendations [1,28,42]. Overall, five *inlA/inlE*, four *inlB,* and three *inlC* allelic variants were detected that correlated with a number of reports on the evident polymorphisms of the internalin proteins [22,24]. There was a certain correlation between the *inlB/inlC/inlE* allele profiles (841/82/177, respectively) and CC1. In fact, this clonal complex was represented by two different STs, namely ST1 and ST734, that were both isolated from an animal source. Additionally, the inlC allele profiles for CC3 (ST3) and ST32 were identical, as the same allele 89 was identified in both *L. monocytogenes,* although these strains were revealed to be from different sources, i.e., food and production environment (Supplemental Data 1).

**Figure 6:**
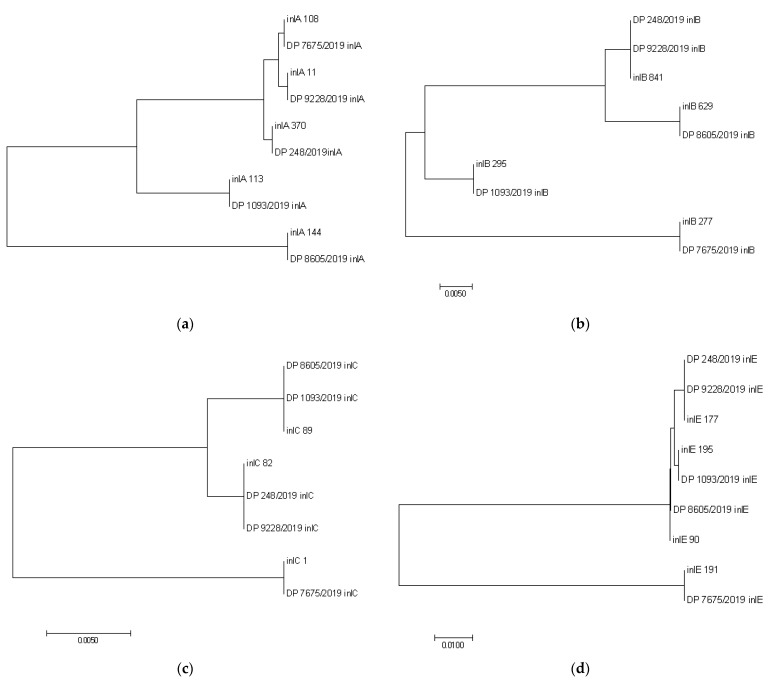
Phylogenetic analysis of the interanalins *inlA*, *inlB*, *inlC,* and *inlE* genes of the *L. monocytogenes* strains isolated in the Republic of Serbia in 2019 and the reference sequences of allelic profiles from the BIGSdb-Pasteur MLST database https://bigsdb.pasteur.fr/listeria/listeria.html, accessed on 22 March 2021). (**a**–**d**) Phylogenetic tree was created in MEGA 7 [30] using the maximum likelihood method with 100% bootstrap support.

The analysis of nucleotide sequences of the internalins *inlA*, *inlB*, *inlC*, and *inlE* genes of the *L. monocytogenes* strains isolated in the Republic of Serbia in 2019 from small ruminants and food products of animal origin did not reveal the presence of single nucleotide polymorphisms (SNPs) related to the reference sequences (Figure 6a–d, Supplemental Data 1). There was a certain identity between the investigated sequences of Serbia-2019 strains and the relevant gene of the reference sequences available in the BIGSdb-Pasteur MLST database https://bigsdb.pasteur.fr/listeria/listeria.html, accessed on 11 June 2021).

Thus, both the MLST and MvLST analysis allowed characterizing the strains isolated from the Republic of Serbia and performing a comparison with modern data. Until now, MLST-based population studies in this territory have mainly been conducted on isolates from human clinical cases in the Republic of Serbia. Our study adds further information on the diversity of *L. monocytogenes* genotypes in small ruminant and food products, as until now strain distribution in animal reservoirs in the Republic of Serbia has not been evaluated. The extension of the present analysis to a higher number of isolates could contribute to a better knowledge of the structure of the *L. monocytogenes* population in this country, in the EU, and worldwide.

## 4. Conclusions

To the best of our knowledge, this study is the first detailed investigation of the polymorphism in the housekeeping genes of the *L. monocytogenes* strains that were isolated in the European country, the Republic of Serbia in 2004–2019. These strains belonged to the phylogenetic lineages I and II and were represented by the ST1, ST3, and ST9 of the clonal complexes CC1, CC3, and CC9, which were common in the majority of the European countries and worldwide, as well as by the novel STs (ST1232, ST1233, ST1234, ST1235, ST1238, ST1236, ST1237, and ST1242) of CC19, CC155, CC5, CC21, CC3, CC315 and CC37, and the rare ST32 (clonal complex ST32) and ST734 (CC1) reported in the Republic of Serbia for the first time. At least four genes of the internalins (*inlA*, *inlB*, *inlC,* and *inlE*) that were earlier found in the more virulent CC1 and CC2 *L. monocytogenes* isolates were successfully detected in Serbia-2019 strains. Importantly, these genes of the internalins were present in the relatively rare sequence type ST32, which does not belong to any of the clonal complexes associated with outbreaks of listeriosis among farm animals and humans. Our study could confirm the association of CC1 with cases of neuroinfection and abortions among small ruminants, and CC3 and CC9 with food products of animal origin, respectively. Given the small number of *L. monocytogene* isolates studied by molecular methods in the Republic of Serbia, large-scale studies are needed to better understand the origin and distribution of virulent strains of this pathogen from different sources, in this country and worldwide.

Therefore, a future strategy should focus on extended multi-laboratory studies applying an appropriate molecular typing technique that allows exchanging data to better understand the origin and distribution of virulent strains of this pathogen in all ecological niches.

## Figures and Tables

**Table 1:** MLST characteristics of *L. monocytogenes* strains isolated in the Republic of Serbia in 2004–2019.

Acc. No in BIGSdb-*Lm* ^1^	Strain ID	Source Category	Sample Origin	ST	CC (MLST)	Phylogenetic Lineage (MLST)	Isolation Year	Source
76379	DP 9228/2019	Animal	Sheep brain	ST1	CC1	I	2019	This study
76380	DP 1093/2019	Food	Cheese with prosciutto	ST3	CC3	I	2019	
76381	DP 8605/2019	Production environment	Fruit sorting machine	ST32	ST32	I	2019	
76382	DP 7675/2019	Food	Smoked pork ribs	ST9	CC9	II	2019	
76383	DP 248/2019	Animal	Aborted goat fetus	ST734	CC1	I	2019	
15906	118/2004	Human	Cerebrospinal fluid	1232	CC19	II	2004	https://bigsdb.pasteur.fr/listeria/, (accessed on 14 December 2016 for 118/2004, 124/2004, 2, 265/2010, 2184/2010, 2960/2012 and 4250/2009; January, 02, 2017 for 22/2008)
15915	124/2004	Human	Cerebrospinal fluid	1233	CC155	II	2004	
15916	2	Food	Fresh rucola-rocket	1234	CC5	I	2008	
16159	265/2010	Human	Cerebrospinal fluid	1235	CC21	II	2010	
16160	2184/2010	Human	Cerebrospinal fluid	1236	CC3	I	2010	
16161	2960/2012	Human	Blood	1237	CC315	I	2012	
16162	4250/2009	Human	Blood	1238	CC21	II	2009	
16122	22/2008	Human	Cerebrospinal fluid	1242	CC37	II	2008	

1 BIGSdb-Lm provides access to genotypic data for *L. monocytogenes* isolates based on multi-locus sequence typing (MLST) (https://bigsdb.pasteur.fr/listeria/, accessed on 11 June 2021).

## Data Availability

Data presented in this study are available upon request.

## Supplementary Materials

The following are available online at https://www.mdpi.com/article/10.3390/microorganisms9061289/s1, Table S1: MLST and MvLST profiles of the *L. monocytogenes* strains isolated in the Republic of Serbia in 2004–2019.
